# Exploring the relationship between safety culture and reported dispensing errors in a large sample of Swedish community pharmacies

**DOI:** 10.1186/2050-6511-13-4

**Published:** 2012-08-13

**Authors:** Annika Nordén-Hägg, Sofia Kälvemark-Sporrong, Åsa Kettis Lindblad

**Affiliations:** 1 Department of Pharmacy, Uppsala University, Box 570, Uppsala S-751 23, Sweden

## Abstract

**Background:**

The potential for unsafe acts to result in harm to patients is constant risks to be managed in any health care delivery system including pharmacies. The number of reported errors is influenced by a various elements including safety culture. The aim of this study is to investigate a possible relationship between reported dispensing errors and safety culture, taking into account demographic and pharmacy variables, in Swedish community pharmacies.

**Methods:**

A cross-sectional study was performed, encompassing 546 (62.8%) of the 870 Swedish community pharmacies. All staff in the pharmacies on December 1st, 2007 were included in the study. To assess safety culture domains in the pharmacies, the Safety Attitudes Questionnaire (SAQ) was used. Numbers of dispensed prescription items as well as dispensing errors for each pharmacy across the first half year of 2008 were summarised. Intercorrelations among a number of variables including SAQ survey domains, general properties of the pharmacy, demographic characteristics, and dispensing errors were calculated. A negative binomial regression model was used to further examine the relationship between the variables and dispensing errors.

**Results:**

The first analysis demonstrated a number of significant correlations between reported dispensing errors and the variables examined. Negative correlations were found with SAQ domains Teamwork Climate, Safety Climate, Job Satisfaction as well as mean age and response rates. Positive relationships were demonstrated with Stress Recognition (SAQ), number of employees, educational diversity, birth country diversity, education country diversity and number of dispensed prescription items. Variables displaying a significant relationship to errors in this analysis were included in the regression analysis. When controlling for demographic variables, only Stress Recognition, mean age, educational diversity and number of dispensed prescription items and employees, were still associated with dispensing errors.

**Conclusion:**

This study replicated previous work linking safety to errors, but went one step further and controlled for a variety of variables. Controlling rendered the relationship between Safety Climate and dispensing insignificant, while the relationship to Stress Recognition remained significant. Variables such as age and education country diversity were found also to correlate with reporting behaviour. Further studies on the demographic variables might generate interesting results.

## Background

The potential for unsafe acts to result in harm to patients is a constant risk to be managed in any health care delivery system. In pharmacies these unsafe acts might consist of dispensing errors that can result in patients receiving the wrong medicine. In community pharmacies, these errors are present in a frequency varying between 0.01% [1,2] and 22% [3], depending on the definition of dispensing errors and the method used to assess these errors. Types of errors include selection errors such as improper choice of medicines, dosage forms, strengths or quantities, as well as erroneous dosage instructions [1,2,4-7]. The causes of dispensing errors vary but commonly noted causes are look-alike packages and similar brand names [1,8]. The context in which these errors occur also have a strong impact and includes such variables as fatigue, high workload, overwork and interruptions [2,5,9].

A variety of measures are used to prevent and manage errors [2,7,9]. One of the main measures is the use of reporting systems, providing possibilities to analyse and subsequently prevent errors. However, research findings show that such structured attempts to collect reports on errors are not always successful and the relationship between actual numbers of errors and the reported number of errors is not clear-cut, since reporting is influenced by a number of elements resulting in lack of reports [10,11]. The reasons include inadequate and unsatisfactory safety procedures, resulting in a lack of common definitions and classification of errors [12], staff ignorance of the purpose of reporting, [13] or shortcomings in staff abilities to follow existing guidelines. [14] They also include the impact of inter- and intra-professional values and interactions. [14] Other reasons can be attributed to the safety culture in the workplace, including employees’ shared perceptions of policies, practices, and procedures that are rewarded, supported and expected [15].

The safety culture is thus an important part of the context, regarding error handling and patient safety issues in health care, including pharmacies., In search for valid yet feasible methods for conducting annual assessments of safety culture, healthcare organisations have used survey questionnaires that measure frontline caregiver perceptions. These provide a snapshot of the larger culture through multiple dimensions such as safety climate, teamwork climate, and stress recognition [16,17].

Studies on the relationship between safety culture and dispensing errors are scarce. In an American study, the overall safety climate of a hospital unit was found to predict medication errors, and a more positive safety culture was associated with fewer incidents [18,19]. A strong safety culture might reinforce adherence to medication administration practices and encourage an open and constructive response to errors [18]. In a strong safety culture, employees tend to perceive procedures as suitable and safety information as available. The norm is to openly confer about safety issues and the willingness to report treatment errors is high [19].

There might be other factors contributing to incidence and reporting of errors. These include demographic variables. Seniority has been found to bring about experience [20], which might reduce the risk for error making. Cultural differences and language difficulties between health care personnel increase the risk for medical misunderstandings [21], which may potentially increase the risk for errors. The term diversity is used to describe the variance of demographic characteristics such as for instance age, education and role at worksite [22]. This aspect, although complex, might add important information about the impact of staff composition on reporting of errors. Pharmacy characteristics may also be related to reported dispensing errors.

The relationship between errors and culture has, to our knowledge, not been systematically studied in community pharmacies. Thus, the aim of this study is to investigate the possible relationship between reported dispensing errors and safety culture, taking into account demographic and pharmacy variables, in Swedish community pharmacies. It has to be pointed out that this is an explorative study only and further analyses on variables might be a next step, given that co-variation is found.

## Methods

A cross-sectional study was performed, using routinely collected pharmacy data and a separately conducted survey distributed to staff at Swedish community pharmacies.

### Setting

Until June 2009 Swedish community pharmacies were owned by the National Corporation of Pharmacies. The corporation was responsible for all of the approximately 870 community pharmacies in Sweden at the time of this study. (Since 2009, a deregulation of the pharmacies in Sweden is in effect, and the pharmacy market has been opened to all interested parties.) There were approximately 7,000 staff members in these pharmacies; the largest professional category was made up of pharmacists (61%) [23].

### Measures

#### Reported dispensing errors

Reporting dispensing errors in Swedish pharmacies is mandatory by law [24]. These reports were, at the time of the study, submitted through a national, web-based error reporting system and kept at the headquarters of the National Corporation of Pharmacies. In December 2007, 14.99 dispensing errors per 100,000 dispensed prescription items were reported in the Swedish community pharmacies [25].

A dispensing error, is a deviation that includes incorrect dispensing, counseling of service to a patient (by the National Corporation of Pharmacies, 2008). This comprises

· Wrong medicine, wrong strength or wrong dispensing form

· Wrong quantity

· Wrong dosage

· Passed expiry date

· Wrong written or verbal information

· Wrong patient or unit

· Missing medicine

· Missing or delayed delivery

· Not noted interaction or double prescribing

Monthly compilations on numbers of reported dispensing errors for each pharmacy from January 2008 until June 2008 were included.

#### The safety attitudes questionnaire

Information on safety culture in Swedish pharmacies was collected using the Safety Attitudes Questionnaire [23]. It is a validated survey instrument that provides a snapshot of staff perceptions, attitudes, and beliefs about quality of safety and teamwork in a particular work setting. The SAQ has six dimensions including Teamwork Climate, Safety Climate, Perceptions of Management, Job Satisfaction, Working Conditions and Stress Recognition [16]. Together these scales provide a multidimensional profile of the safety-related norms in a given work setting. Higher scores on each of these scales, represent more safety awareness and readiness to manage risk by the staff.

All the people listed as employed in all Swedish community pharmacies on December 1^st^, 2007 were asked to participate in the survey on safety climate; SAQ. The survey was translated and adapted for use and distributed to staff in Swedish community pharmacies in 2008 [23].

#### Demographic variables

Respondent demographic items included age, country of birth, educational level as well as in which country the education was provided, and role in pharmacy (e.g. pharmacy manager) [23].

#### Dispensed prescription items

Numbers of dispensed prescription items; DPIs, were available from the National Corporation of Pharmacies. These data were compiled for each pharmacy from January through June of 2008. Inclusion criteria for pharmacies, based on volume, included only pharmacies with at least 1,000 dispensed prescription items during this period. Only one pharmacy had less than 1,000 DPIs^a^ and was hence excluded in this study.

#### Response rate

Response rate was studied as an extra control variable in order to investigate if general responsiveness among the staff had an impact on the possible relationship between safety climate and dispensing errors.

### Study group

Pharmacies with at least three respondents were included. Out of the total number of pharmacies 546 (62.8%), including 3,654 (54.7%) respondents, met the inclusion criteria of at least three respondents and 1,000 dispensed prescription items during the first half year of 2008.

The SAQ is originally validated for units with at least five respondents [16]. The rationale behind this threshold was to protect the confidentiality of respondents and to target a minimum number of individuals to assess a culture [26]. However, a considerable number, approximately 27%, of Swedish pharmacies have three or less employees. Allowing the use of lower threshold of respondents per pharmacy would meaningfully increase the usability of this survey tool. Consequently, the validity of a lower threshold of respondents in pharmacies was tested, under the assumption that a unit with at least three individuals may also have a joint culture. The psychometric validation of this group of respondents is included in Additional file 1: Appendix A.

### Statistics

#### Level of analysis

The analysis was conducted at the pharmacy level. Individual questionnaire responses were aggregated by calculating, for each pharmacy, the mean scores of each variable. The SAQ uses consensus assessments whereby group-level perceptions are garnered to see what views the pharmacy personnel have in common [27-29]. To justify the aggregation of scores from the individual to the pharmacy level of analysis, homogeneity of scores or a within-unit agreement and between-unit variance should be demonstrated. James, Demaree, and Wolf’s r_wg(j)_ index [30] was computed; this is a measure of intra-group agreement of homogeneity. The r_wg(j)_ agreement index represents the interchangeability of respondents and is used to determine the appropriateness of aggregating data to higher levels of analysis. It attempts to determine whether one group member’s response is basically identical to another group member’s response. The r_wg(j)_ is a group-specific index; that is, it is an index that is calculated for each of the groups in the sample. Any r_wg(j)_ values greater than 0.70 are viewed as providing acceptable support for aggregating data to a unit level of analysis [31].

ICC(1) (Intraclass Correlation Coefficient) values represent the amount of variance in individual perceptions that can be explained by unit or team membership; i.e. being a staff member in a specific pharmacy. ICC(2) is an index that represents the reliability of the group mean within a sample and varies as a function of group size and the ICC(1) value. ICC(1) was computed from a one-way ANOVA. In this ANOVA the SAQ dimensions comprise the variable of interest (dependent variable) and pharmacy membership is the independent variable [31]. ICC(2) was computed from ICC(1) via the Spearman-Brown formula [31]. Many researchers simply evaluate the statistical significance of the ICC(1) value to assess whether there is meaningful non-independence among survey responses [32,33] which is also done in this study. Together, this package of indices gives insight into how much the members of a pharmacy agree with one another and how different teams are from one another, both of which are important for understanding the impact of combining individual team member perceptions into team-level metrics. The analyses were carried out using functions provided in the multilevel package for R; version 2.10.0, 2010.

The result of the r_wg(j)_ analyses is included in Additional file 1. The r_wg(j)_ agreement index presented for the SAQ domains shows moderate (Stress Recognition, Perceptions of Management), but mainly strong agreement within pharmacies (Table 1). ICC(1) values were all statistically significant, demonstrating between-unit significance for all survey domains. However some variation was present, and while 19% of the variability in any one respondent’s rating of Teamwork Climate is a function of the pharmacy group to which the individual belongs, only 4% of Stress Recognition is a function of this group belonging. The ICC(2) values for Job Satisfaction and Perceptions of Management are reasonable. Acceptable within-unit homogeneity was however present across survey domains, with the exception of the Stress Recognition domain. In the case of Stress Recognition, there is significant variability between pharmacies, but relative to the other scales, the source of variation coming from within the pharmacy as a *collective view* was lower. This suggests that Stress Recognition is less of a consensus perception than the other domains, which is consistent with previously published studies [16,34]. Thus Stress Recognition might be considered as an additive construct [29].

**Table 1:** **Aggregation metrics for team-level consensus composition constructs**^ab^

	**ICC(1)**	**ICC(2)**	$\bar{X}$**r**_**wg(j)**_	**SD r**_**wg(j)**_
Teamwork Climate	0.19**	0.58	0.82	0.26
Safety Climate	0.15**	0.50	0.88	0.18
Job Satisfaction	0.22**	0.68	0.83	0.25
Stress Recognition	0.04**	0.21	0.68	0.32
Perceptions of Management	0.23**	0.65	0.70	0.27
Working conditions	0.16**	0.47	0.74	0.25

^a^ Individual N = 3,654; Pharmacy N = 546.

^b^ *p < .05, **p < .01 two-tailed.

#### Data analysis

In a descriptive analysis, intercorrelations for all the variables in the questionnaire, as well as number of employees per pharmacy, dispensed prescription items per pharmacy, response rate and errors were calculated using R.

Based on these intercorrelations, a negative binomial regression model was used to further examine the relationship between pharmacy characteristics and domains of the SAQ and the outcome dispensing errors. This model is appropriate when modelling a non-zero, count-based outcome in which there is overdispersion [35]. Functions in the MASS package of R were used to estimate the negative binomial models. The results are to be interpreted as follows: For a one unit change in the predictor variable, i.e. the difference in the logs of expected counts of the response variable is expected to change by the respective regression coefficient, holding all other variables constant.

### Approval of ethics committee

No approval was required from the ethics committee according to the Swedish law^b^ at the time of the data collection. Ethical considerations were met however; responding to the questionnaire was voluntary and all answers were de-identified to maintain confidentiality.

## Results

In the descriptive analysis the means, standard deviations, and correlations among the variables at the pharmacy were calculated (Tables 2 and 3). A number of significant correlations between dispensing errors and SAQ dimensions were found. A significant negative correlation was found between dispensing errors and Teamwork Climate (−0.09), Safety Climate, (−0.12) and Job Satisfaction (−0.12) respectively; high levels in these SAQ dimensions were associated with low levels of errors. A significant positive relationship was demonstrated between the Stress Recognition dimension (0.10) and dispensing errors, i.e., respondents that acknowledged the impact of stress on their performance, were more likely to report dispensing errors.

**Table 2:** **Pharmacy-Level means, standard deviations and intercorrelations of SAQ dimensions and dispensing errors**^abc^

	**M**	**SD**	**Teamwork Climate**	**Safety Climate**	**Job Satisfaction**	**Perceptions of management**	**Working Conditions**	**Stress Recognition**
Teamwork Climate	4.42	0.46	(0.90)					
Safety Climate	4.28	0.38	0.79**	(0.87)				
Job Satisfaction	4.32	0.50	0.75**	0.74**	(0.92)			
Perceptions of Management	3.81	0.55	0.59**	0.61**	0.63**	(0.85)		
Working Conditions	3.88	0.52	0.58**	0.62**	0.57**	0.63**	(0.78)	
Stress Recognition	3.88	0.45	−0.09*	−0.12**	−0.16**	−0.26**	−0.18**	(0.74)
Dispensing errors	6.35	5.82	−0.09*	−0.12**	−0.12**	−0.06	−0.07	0.10*

^a^ Pharmacy-level N = 546.

^b^ For correlations |0.09|, p < .05; |0.11|, p < .01.

^c^ Pharmacy-level inter-item reliability (i.e., Cronbach’s α) for multi-item scales is in parentheses along the diagonal.

**Table 3:** **Pharmacy-level means, standard deviations, and íntercorrelations of pharmacy characteristics and dispensing errors**^**ab**^

	**M**	**SD**	**1**	**2**	**3**	**4**	**5**	**6**	**7**	**8**	**9**	**10**
1 Number of employees	6.69	3.77	-									
2 Mean age	49.85	5.66	−0.05	-								
3 Mean education	2.20	0.26	0.13**	0.06	-							
4 Age diversity^1^	10.25	3.69	0.14**	−0.45**	−0.06	-						
5 Education diversity^1^	0.43	0.19	0.37**	−0.12**	0.26**	0.10*	-					
6 Birth country diversity^2^	0.15	0.21	0.12**	−0.23**	−0.13**	0.09*	0.24**	-				
7 Education country diversity^2^	0.08	0.15	0.12**	−0.17**	−0.16**	0.05	0.28**	0.71**	-			
8 Role diversity^2^	0.53	0.13	0.06	0.03	0.38**	−0.04	0.50**	0.02	0.06	-		
9 Response rate	66.68	21.56	0.06	0.04	0.08*	0.02	−0.03	−0.06	−0.04	0.15**	**-**	
10 DPI^3^	50276.59	26562.48	0.79**	−0.08	0.03	0.10*	0.32**	0.16**	0.14**	−0.03	−0.39**	-
11 Dispensing errors	6.35	5.82	0.53**	−0.11*	−0.01	0.07	0.25**	0.19**	0.20**	−0.02	−0.26**	0.64**

^a^ Pharmacy-level N = 546.

^b^ For correlations |0.09|, p < .05; |0.11|, p < .01.

^1^ Age diversity and Education diversity is an assessment of Standard Deviation.

^2^ Birth Country Diversity, Education Country Diversity and Role Diversity is calculated using Blau’s index; an index to measure variety across categories. It ranges from 0 to 1, with 1 indicative of more variety in a given grouping [36].

^3^ Dispensed Prescription Items.

Reported errors were significantly positively correlated to number of employees, educational diversity (i.e. a higher value indicates greater variety across pharmacy members in their education background), birth country diversity, education country diversity, and number of dispensed prescription items. Thus pharmacies with higher numbers of reported dispensing errors were also likely to have a high number of staff, a diverse staff (education level/country of education/country of birth) and also, a high number of dispensed prescription items. A significant, but negative, correlation was found between reported dispensing errors and mean age, i.e. the older the staff the lesser the numbers of dispensing errors. A negative correlation was also demonstrated between response rates and reported dispensing errors; pharmacies with high response rates on our survey demonstrated fewer dispensing errors.

A second analysis was carried out; i.e. those variables displaying a significant relationship to reported dispensing errors in the descriptive analysis, were included in a negative binomial regression analysis, displayed in Table 4. The number of dispensed prescription items and number of employees were both significantly and positively related to reported dispensing errors. Mean age was significantly and negatively related to these errors. Pharmacies that were more diverse with respect to whether staff members had received their education outside Sweden tended to report more errors. When controlling for respondent demographics, the only SAQ survey domain significantly related to dispensing errors was Stress Recognition; pharmacies in which respondents reported higher levels of stress recognition had higher frequencies of reports on dispensing errors.

**Table 4:** **Results of negative binomial regressions predicting number of dispensing errors**^abc^

	**1**	**2**	**3**	**4**
Intercept	1.71	1.71	1.70	1.70
Number of DPI^1^	0.01**	0.01**	0.01**	0.01**
Number of employees	0.05**	0.06**	0.05**	0.05**
Response rate	−0.01**	−0.01**	−0.01**	−0.01**
Mean education		−0.10	−0.12	−0.07
Mean age		−0.01	−0.01*	−0.01*
Education diversity			0.34	0.31
Education country diversity			0.47*	0.47*
Age diversity			−0.01	−0.01
Teamwork climate				0.09
Safety climate				0.12
Job satisfaction				−0.15
Perceptions of management				0.12
Working conditions				−0.15
Stress recognition				0.19*
AIC	2916.90	2913.7	2906.2	2836.20

^a^ Pharmacy N = 546.

^b^ *p < .05, **p < .01 two-tailed.

^c^ All predictor variables are mean- centred.

^1^Dispensed prescription items.

## Discussion

This study explores the relationship between safety climate and the reporting of dispensing errors in a national sample of community pharmacies in Sweden. An association between safety climate and errors has been established in other parts of health care [18,19]. No significant relationship between reported dispensing errors in Swedish community pharmacies and Safety Culture, after controlling for variability in respondent and pharmacy demographics, was found. The presence of an unusually strong safety culture in these community pharmacies, as compared to other health care settings in the USA [23], has been previously reported. An explanation for this strong culture might be the fact that the National Corporation of Pharmacies for a long time put great effort into quality management and worked intensively on initiating measures for continuous improvements [37]. This included elements like definite guidelines; i.e. standard operation procedures for the dispensing process and other processes. Various indicators were used to assess quality in pharmacies and for instance all staff went through quality education around 2000. Thus it could be assumed that good quality awareness, with a ruling influence on safety issues in pharmacies, was present and impacted the outcome of this survey.

Thus one possible explanation for the lack of association is that a ceiling effect may have reduced the possibility to discriminate between pharmacies. Anecdotally, recent work at Johns Hopkins Hospital suggests that the more mature a reporting system is, the more the relationship between SAQ dimensions and error reporting declines [38]. Perhaps it is the case that, as staff build confidence and trust around safety standards and reporting procedures, the predictive power of safety culture as a proxy for “safety-related trust” is diminished. The system becomes a natural part of the work place and therefore only an increasingly weak relationship with reported dispensing errors would be found, which could be one explanation to the pattern of results found in the current study. The differences between settings in this study compared to those in the other studies; i.e. hospital units vs. pharmacies, as well as difference in instruments used for assessing safety climate and error-reporting systems used, also make direct comparisons difficult. As our study is larger than the other studies, lack of power is however not likely to explain the lack of association, if there is one.

The SAQ dimension Teamwork Climate has also been demonstrated to be strong in Swedish community pharmacies, [23] and presumed to reveal prevalence of good co-operation and respect among staff [39,40]. As already noted, no relationship was found with dispensing errors in this study, after controlling for demographic variables. Again, a ceiling effect might partially explain this.

The only Safety Attitudes Questionnaire domain that was significantly, positively, correlated with dispensing errors, after controlling for demographics, was Stress Recognition. In SAQ this dimension is an indicator of individual attitudes rather than of group attitude, since the dimension, unlike all other dimensions, is dominated by items referring to “I” rather than “we” (see Additional file 1). It might be questioned whether there is a place for a dimension primarily assessing individual’s self-awareness within the framework of the presumed collective safety climate area. The within-unit and between-unit analysis has however ensured that this variable performs satisfactorily at group level, although considerably poorer than the other dimensions. When staff members in a pharmacy experience dispensing errors, the awareness of the risk of errors may increase, with increased stress recognition among staff as one possible outcome. This may explain the counterintuitive relationship between stress recognition and dispensing errors, where more self aware staff members, with regard to how they behave under pressure, is associated with more reported dispensing errors. This seems to be contrary to prior research linking higher stress recognition to better performance in commercial aviation pilots [41], but further investigation is warranted. In an American study, safety climate was negatively related to incident reporting volume, while stress recognition was independently positively related to incident reporting volume, which correlates with our findings [42] The difference between that study, and the current study, is that this national sample of community pharmacies included far more demographic variables, which were not controlled for in the American study. If controlling for demographic variables diminishes the predictive power of safety culture over incident reporting, then the current study has identified the importance of controlling for respondent and site demographic variables. It is possible that the size of this nation-wide study was so large, and the number of demographic variables was so comprehensive, that few other studies (to date) into incident reporting have the ability to attempt such an analysis.

Relationships were found between high levels of dispensing errors and high numbers of dispensed prescription items and employees, respectively. This might be an indication of the fact that the bigger the pharmacy, in terms of number of employees and prescription volumes the busier the surroundings are. It might become difficult to convey information on safety issues and prescriptions and have informative communication between colleagues; misunderstandings might be more common. It will also become harder to get to know your colleagues [43].

A relationship was also found between age and dispensing errors; the higher the mean age in a pharmacy is, the lower the number of dispensing errors is. Seniority has been found to bring about experience [20]. The senior staff might make fewer errors, as they are more experienced, know the pitfalls and can avoid them. Who makes most errors – the experienced staff or the more junior staff? This question has been evaluated by O’Shea [44] in a literature review, but the answer was inconclusive.

In the first correlation analysis a number of relationships regarding demographic diversity were found and significant relations were found between reported errors and education, birth country as well as education country. The only remaining relationships, after having controlled for covariates in the regression analysis were education background diversity and an association between having a heterogeneous staff with regard to educational background (non-Swedish/Swedish) and dispensing errors. The more multifaceted the educational background is, the more errors are reported. Misunderstandings between different cultural groups of health care personnel have been reported in Sweden [21]. Cultural differences and language barriers in pharmacies might lead to misunderstandings and misinterpretations, resulting in more errors. A non-native health-care staff might also experience a more difficult working situation in relation to patients, due to cultural differences [45] and communication problems [46] which might increase the risk for errors. It is important, however, to remember that these problems are balanced by the advantages of having multicultural competence at the working site and the degree of advantages depends largely on leadership [47]. This exploration suggests a possible relationship between demographic diversity variables and reported errors. The theory behind demographic diversity is complex [22] and an in-depth analysis might be worthwhile.

A negative association was found between the numbers of dispensing errors and response rate. A high response rate on a questionnaire about safety attitudes might be a measure of the staff’s attentiveness to these issues. If so, a high response rate might be an indicator of responsible behaviour, which in turn might be associated with deliberate and careful dispensing behaviour.

A high agreement between reported errors and actual errors is assumed, based on the fact that the reporting system is relatively mature [23]. The Swedish reporting system is now over 10 years old and administrative procedures are in place. There is a clear-cut definition of a dispensing error and specific guidelines regarding handling of errors. Such clarity is considered to positively incentivize reporting behaviour [12,14]. Several measurements have been made over the years, which has put a focus on dispensing errors in the National Corporation of Pharmacies, e.g. the introduction of an intervention, targeted to reduce specific errors [22]. Feed-back has been provided to the users on a regular basis over the years. Other studies have demonstrated that when safety climate is very positive (i.e. safety “trust” is high), the reported number of errors is closer to the actual number of errors [48]. Experiences of previous handling of errors influence the way staff behave, i.e. a mature and non-punitive approach to errors will result in a higher degree of detecting and reporting of errors.

## Conclusion

This study replicated previous work linking safety climate to reporting behaviour, but went one step further and controlled for a variety of demographic variables. After controlling these variables, the relationship between safety climate and dispensing errors was rendered insignificant, while the relationship to stress recognition remained significant. A few demographic variables; i.e. age and education country diversity also were found to impact reporting behaviour. Further studies on the demographic variables might generate interesting results.

## Endnotes

^a^This pharmacy was judged either to have very limited opening hours or to be in the process of closing.

^b^http://www.riksdagen.se/sv/Dokument-Lagar/Lagar/Svenskforfattningssamling/Lag-2003460-om-etikprovning_sfs-2003-460/ [Swedish only]. The law state that ethical approval is needed if: 1. the research involves storing sensitive personal data 2. The research involves storage of data on crime and sentences 3. If there is an intended physical or psychological impact from the research (e.g. clinical trials of medicine, testing new therapies) and 4. The research involves tissue from humans. None of this is applicable on this research. No data was stored that could link an answer to a specific individual.

## Authors' contributions

ANH - Initiating project, planning project, acquisition of data, analysis and interpretation of data, drafting of manuscript, revising manuscript, final approval. SKS - Planning project, analysis and interpretation of data, revising manuscript, final approval. AKL - Planning project, analysis and interpretation of data, revising manuscript, final approval. All authors read and approved the final manuscript.

## Competing interests

Annika Nordén-Hägg and Sofia Kälvemark Sporrong were, at the time of planning and data collection, employed by the National Corporation of Swedish Pharmacies.

Åsa Kettis has no competing interests.

## Pre-publication history

The pre-publication history for this paper can be accessed here:

http://www.biomedcentral.com/2050-6511/13/4/prepub

## Supplementary Material

Additional file 1 Appendix A. Click here for file
