# Supplementary material for: Exploring the relationship between safety culture and reported dispensing errors in a large sample of Swedish community pharmacies
Source: BMC Pharmacol Toxicol. 2012 Aug 13;13:4. doi: 10.1186/2050-6511-13-4 (PMC3506269; doi:10.1186/2050-6511-13-4)
Supplement: Additional file 1 — Appendix A. [file 2050-6511-13-4-S1.docx]

Appendix A

# The SAQ is originally validated for units with at least five respondents. ^16^ The rationale behind this threshold was to protect the confidentiality of respondents and to target a minimum number of individuals to assess a culture ^27^. However, a considerable number, approximately 27%, of Swedish pharmacies have three or less employees. Allowing the use of lower threshold of respondents per pharmacy would meaningfully increase the usability of this survey tool. Consequently, the validity of a lower threshold of respondents in pharmacies was tested, under the assumption that a unit with at least three individuals may also have a joint culture.

# Psychometric validation of respondent group

The psychometric properties of the group of pharmacies included in this study were examined by analysing the latent factor structure of the survey items. The inter-item reliability of the scale domains was examined as well, using Cronbach’s alpha. Individual survey respondents are grouped within pharmacies and therefore a multilevel confirmatory factor analysis was performed, accounting for the statistical non-independence of observations within pharmacies. ^46^ The hypothesized factor structure of the SAQ was modelled, consistent with prior research, ^16^ at both the within- and between-levels of analysis, using M*plus* Version 5.2. ^47^

The study group included 546 community pharmacies (3,654 respondents), fulfilling the criteria of at least three respondents and at least 1,000 dispensed prescription items during the first half year of 2008. The psychometric validation of the SAQ in this group demonstrated a moderate fit of the data to the hypothesized model. This result was probably explained in part by low intra-class correlations for a number of survey items (table 7). Moreover, a few survey items did not lead strongly on their hypothesized factors. As stated already in the original psychometric validation ^25^ the negatively worded items “If I detect problems with medication management, it is difficult to address this, in this pharmacy.” and “In this pharmacy, it is difficult to discuss mistakes” were supplemented by positively worded versions of the same questions. The negative versions of these items did not work well with respect to either factor structure or inter-item reliability. Instead the positively worded versions were included (table A1; items 2 and 11). The diagonal values in parentheses in table 4 provide Cronbach’s alpha for each of the multi-item survey domains.

**Table A Swedish Community Pharmacy version: Items and Factor Loading ^a b c^**

|  | | **Within-Level** | **Between-Level** |
| --- | --- | --- | --- |
| ***Teamwork Climate*** | | | |
| 1. | My views and suggestions – my in put – is well received in this pharmacy. | 0.63 | 0.99 |
| 2. | If I detect problems with medication management, it is easy to address this, in this pharmacy. (The term “medication management” refers to all activities have to do with medication, such as product management and expediting of both prescriptions and over-the –counter products) | 0.50 | 0.86 |
| 3. | If we in the staff do not agree in a matter, it will be resolved in a positive way (i.e. our concern Is not who is right, but rather what is best for the customer) | 0.72 | 0.90 |
| 4. | I receive the support I need from other staff in this pharmacy to help the customers. | 0.73 | 0.99 |
| 5. | It is easy for staff members here to ask questions when there is something they don’t understand. | 0.74 | 0.99 |
| 6. | The staff members at this pharmacy work together as a well-functioning team. | 0.74 | 0.98 |
| ***Safety Climate*** | | | |
| 7. | I would feel safe as a customer here. | 0.50 | 0.63 |
| 8. | Dispensing errors are handled in a correct way at this pharmacy. | 0.50 | 0.40 |
| 9. | I know how to appropriately direct questions regarding patient safety in this pharmacy. | 0.54 | 0.65 |
| 10. | I receive constructive feedback for the work I do. | 00.68 | 0.80 |
| 11. | In this pharmacy it is easy to discuss mistakes. | 0.68 | 0.98 |
| 12. | I am encouraged by others in this pharmacy to speak about any thoughts I have regarding patient safety. | 0.63 | 0.99 |
| 13. | The work environment in this pharmacy makes it easy to learn from the mistakes of others. | 0.68 | 0.90 |
| ***Job Satisfaction*** | | | |
| 14. | I like my work. | 0.59 | 0.55 |
| 15. | Working here is like being a part of a large family. | 0.76 | 0.95 |
| 16. | This pharmacy is a good workplace. | 0.90 | 0.98 |
| 17. | I am proud that I work at this pharmacy. | 0.83 | 0.94 |
| 18. | There is a good atmosphere at this pharmacy. | 0.79 | 1.00 |
| ***Working conditions*** | | | |
| 19. | This pharmacy does a good job of training new staff members. | 0.65 | 0.60 |
| 20. | I normally have access to the information needed for judging whether a prescription is reasonable. | 0.49 | 0.71 |
| 21. | In this pharmacy newly employed staff is under adequate supervision. | 0.68 | 0.65 |
| 22. | Problematic staff members are handled in a constructive way by the management of this pharmacy. | 0.63 | 0.95 |
| ***Perceptions of Management*** | | | |
| 23. | The management of this pharmacy supports me in my daily work. | 0.77 | 1.00 |
| 24. | The management of this pharmacy would not consciously endanger patient safety. | 0.55 | 0.91 |
| 25. | The management of this pharmacy provides me, sufficiently in advance, with necessary information about events that could affect my work. | 0.66 | 0.80 |
| 26. | The staff level at this pharmacy is sufficient to handle the number of customers. | 0.50 | 0.47 |
| ***Stress Recognition*** | | | |
| 27. | When my workload gets too heavy, my performance gets worse. | 0.65 | 1.00 |
| 28. | I am less efficient at work when I am tired. | 0.80 | 0.89 |
| 29. | I am more likely to make mistakes in stressful or unpleasant situations. | 0.74 | 0.64 |
| 30. | Fatigue impairs my performance in trying situations. | 0.91 | 0.81 |

^a^ Individual N = 3.654; Pharmacy N = 546

^b^ CFI = 0.92; RMSEA = 0.04

^c^ Loadings are standardized path coefficients from a multilevel confirmatory factor analysis of the items on the hypothesized factors
